# Development and implementation of an integrated orthoptic–speech therapy assessment protocol in neurorehabilitation setting: A community case study

**DOI:** 10.3389/frhs.2026.1715851

**Published:** 2026-05-20

**Authors:** Rosaliria Grossi, Federica Guidi, Francesca Rossi, Mariangela Vaira, Patrizia Cancialosi, Lucia Portis, Maurizio Beatrici, Lorella Cocchini, Chiara Maria Eandi

**Affiliations:** 1 Department of Mental Health, ASST Pavia, Pavia, Italy; 2 Department of Clinical, Surgical, Diagnostic, and Pediatric Sciences, University of Pavia, Pavia, Italy; 3 Department of Surgical Science, Eye Clinic, AOU “Città della Salute e della Scienza di Torino”, Turin, Italy; 4 Department of Surgical Sciences, University of Turin, Turin, Italy; 5 Department of Rehabilitation Service, Public Health and Paediatric Sciences, AOU “Città della Salute e della Scienza di Torino”, Turin, Italy; 6 Department of Public Health and Paediatric Sciences, University of Turin, Turin, Italy; 7 SC Neurorehabilitation, AOU “Città della Salute e della Scienza di Torino”, Turin, Italy; 8 Department of Medical Sciences, University of Turin, Turin, Italy; 9 Department of Ophthalmology, University of Lausanne, Fondation Asile des Aveugles, Jules Gonin Eye Hospital, Lausanne, Switzerland

**Keywords:** acquired brain injury, action research, ICF, interdisciplinary evaluation, multidisciplinarity, neurorehabilitation, orthoptist, speech and language therapist

## Abstract

**Introduction and context:**

Patients with Severe Acquired Brain Injuries (SABI) are a complex challenge in neurorehabilitation. They often cause visual and cognitive-communicative disorders that require a multidisciplinary approach, yet few clear strategies exist for effectively integrating rehabilitation professionals and optimizing care pathways. To improve care for complex patients, this study aimed to examine how clinicians perceive the impact of deficits in vision and communication. The research specifically focused on the state of team collaboration among clinicians (including physicians and nurses), healthcare professionals, and between speech and language therapists and orthoptists, while identifying effective strategies to address these challenges.

**Methods:**

This qualitative study used a mixed-method approach, combining documentary research, participant observation, a focus group, and an open-ended questionnaire, which were administered to two distinct groups of professionals. Data were collected and triangulated to ensure ecological rigor and provide a deep understanding of the clinical process and its challenges. All data were analyzed thematically to identify recurring themes and common perceptions.

**Findings and discussion:**

The results confirmed clinicians' perception of the important role that visual, cognitive, and communicative disorders play. They highlight a perceived lack of information regarding visual aspects in medical records. The analysis revealed a clear difference in the perception of professional integration; the Speech and Language Pathologist was perceived as a well-integrated figure, while the orthoptist was found to be less effective in the team. The participants' recommendations emphasized the need for shared written evaluation communication tools regarding speech and language therapy and orthoptic aspects. These tools should use a common language, such as a written protocol.

**Conclusions:**

The study underscores the importance of overcoming informational and coordination barriers between disciplines to optimize the care pathway for patients with SABI. The results suggest that the adoption of a shared protocol can significantly improve multidisciplinary integration and the quality of care.

## 1 Introduction

Severe Acquired Brain Injury (SABI) is an acute, non-congenital, perinatal, or degenerative brain damage resulting in a coma (Glasgow Coma Scale 8 for more than 24 h). It leads to complex and multiple sensory-motor, cognitive, linguistic, and behavioral impairments. And it is often responsible for severe and persistent disability (1). The care pathway requires an acute hospital phase followed by intensive rehabilitation in specialized units (2, 3). Even after hospitalization, outcomes requiring healthcare and social interventions for family, social, school, and work reintegration, often persist after discharge, making the process of care being a highly complex task on both a clinical and organizational level (1).

Despite the importance of holistic, multidisciplinary evaluation (1, 3–8), full clinical integration remains challenging. Although visual impairments are prevalent in SABI patients (4, 9–11), specialized assessments are scarce and Ophthlmological rehabilitation is delayed or even absent. Similarly, in-depth evaluation of communication and language is often compromised by the absence of a single, comprehensive tool to assess all Executive Functions (EFs), including language (6). Such visual deficits significantly impact autonomy and functional recovery (8, 12). However, ophtalmic-orthoptic aspects can also serve as objective indicators of EFs, complementing traditional cognitive tests that may be affected by patients' communication difficulties (13).

The lack of communication and information integration among professionals, aggravated by the complexity of clinical pictures, compromises clinical, research and managerial aspects (14).

Currently, no specific clinical-organizational processes or tools addressing these issues are available in the literature.

From an initial comparison with the working group and the literature, the need to develop an evidence-based, ICF-based protocol and to be applied in the hospital context emerged. This tool would evaluate cognitive, communicative, visual, and linguistic disorders in a person with SABI, possibly mediated by computer resources (15), to facilitate the optimization of the care process. This would fill a significant gap in both the scientific and gray literature, where similar solutions are not present.

In the Neurorehabilitation department of the University Hospital “Azienda Ospedaliera Universitaria, Città della Salute e della Scienza di Torino” (AOU), orthoptic-ophthalmological consultation is available internally. However, there is an opportunity to enhance its effectiveness through a reorganization of departmental activities and the implementation of standardized procedures.

Given the significant impact of such deficits on a person's care, the ultimate goal of this project is to enhance multidisciplinary care in a Neurorehabilitation department by integrating the orthoptist, a healthcare professional who treats motor and sensory visual disorders and performs instrumental-ophthalmological semiology techniques. The project focused on three main objectives: 1. to understand the dynamics, challenges and opportunities of a multidisciplinary rehabilitation approach and what can be made more efficient, with particular attention to the strengths and weaknesses of the current collaboration; 2. to identify possible strategies to improve the current rehabilitation pathway (such as Joint Orthoptic-Speech Therapy Protocol); and 3. to evaluate the perceived effectiveness of at least one of the outlined strategies.

## 2 Context

### 2.1 Setting and population

The Community Case Study context was the AOU Neurorehabilitation department, one of the main centers for the management of SABI in Piedmont.

Study participants were selected from the AOU team via purposive, non-probabilistic sampling among staff with at least one year of experience in the Neurorehabilitation and Ophthalmology services; sampling was delegated to the clinical area coordinators.

To ensure data saturation and clinical representation without disrupting hospital activities, the recruitment of professionals was planned, including at least one representative for each rehabilitative discipline present in the department (SLP, nurse, medical specialist in Physical Medicine and Rehabilitation, and orthoptist). The presence of two physiotherapists was requested, due to their prevalence in the unit. The presence of 7 participants was expected, with 85% participation considered acceptable.

To ensure greater transferability and external validity, professionals from other Italian Neurorehabilitation departments identified as state-of-the-art in the field of SABI through institutional websites and scientific publications, were also involved; experts were invited to participate and to recruit further knowledgeable colleagues. Participant recruitment was conducted until data saturation was reached, with the collection of 25–50 interviews estimated *a priori* (16, 17).

## 3 Description of the intervention

The Community Case Study adopted a qualitative approach, combining an Action Research (AR) design with ethnographic elements. The methodology was structured into two main phases: an observational phase and a development and pilot phase.

Data collection employed a triangulation of methods and spaces to improve the study's validity and transferability. It was structured in:

### 3.1 Observational phase

This phase focused on identifying clinical challenges and staff perspectives within the Neurorehabilitation department. Through direct contextual engagement, we identified three main care process barriers and proposed corresponding solutions (Table 1). Between 2022 and 2023, for this purpose, we employed: a comprehensive situational analysis through literature reviews, documentary research, Participant Observation (PO) (Supplementary Materials S1, S2), Focus Group (FG) in the AOU Neurorhiabilitation (Table 1), and Open-Ended Computerized Questionnaire (OEQ) electronically to professionals from other Italian Neurorehabilitation departments (Tables 1, 2).

**Table 1:** The table illustrates the methodological articulation of the initial phase of action research. It explicitly details the connections between the study's objectives, the actions undertaken, and the tools or methods used, and the indicators for measuring the achievement of each set objective are specified. Furthermore, the table explains specific methods from ethnographic studies to pursue the criteria of credibility, transferability, dependability and confirmability.

Study Objective and Outcome Indicator	To evaluate the degree of involvement, perspectives, and challenges of the department staff	
Action	Tool or Method	Process/Outcome Indicator
Collection of information and data concerning internal procedures and dynamics within the department	Participant observation Documentary collection	To specify, at least: Phases of multidisciplinary evaluation Procedures and tests used by the department
Study Objective and Outcome Indicator	Statement of at least three strategies to promote collaboration among the various professional figures and the implementation of at least one of them	
Action	Tool or Method	Process/Outcome Indicator
Promotion of sharing experiences and perspectives of Italian professionals	Focus Group	Achieving at least 85% participation of invited staff in the focus group Counting at least 7 interventions per profession Statement of at least 3 strategies to promote collaboration
	Questionnaire	Participation of at least 3 professionals from each external facility Perception of the orthoptist's role: at least 3 positive and 3 negative aspects to address
Study Objective and Outcome Indicator	Experimentation of the identified strategy, with the definition of at least three strengths by the working group.	
Action	Tool or Method	Process/Outcome Indicator
Drafting of the evaluative protocol considering the strategies and experiences provided by the professionals involved	Study products	Implementation of at least 1 possible solution
Techniques for Improving Reliability		
**Credibility:** The field observation phase allowed for familiarity with the context and the creation of bonds, favoring a greater quantity of data and an in-depth understanding. The analysis was conducted independently by multiple researchers, and the results were discussed and compared to verify consistency. Furthermore, member checking was performed, by comparing the emergent content with the participants themselves to validate the accuracy of the observations.		
**Transferability:** In addition to the use of triangulation, attention was paid to collecting sufficient data to outline the department's context and the participants’ experiences, allowing for a detailed description of the studied reality.		
**Dependability and Confirmability:** Efforts were made for a transparent study that conformed to the qualitative methodology, including the use of two checklists: SRQR (Standards for Reporting Qualitative Research) (O'Brien et al., 2,014) for the methodological framework of the qualitative component; SQUIRE 2.0 (Standards for QUality Improvement Reporting Excellence) (Ogrinc et al., 2015) for the overall structure and description of the intervention.		

**Table 2:** The table shows the open-ended computerized questionnaire sections.

Section Number	Section Topic
Section 1	General information: age, profession, education, workplace, years of experience.
Section 2	Perception of visual/cognitive-linguistic disorders: pairs of dichotomous/open-ended questions on the interference of these deficits in the care pathway.
Section 3	Professional integration: perception of the integration of physical therapists, speech-language pathologists, and orthoptists in the multidisciplinary team, including strengths and weaknesses.
Section 4	Attention to areas of the Individualized Rehabilitation Project (IRP) and propensity for teamwork: pairs of questions on the inclination toward group work and the use of a common tool for the assessment of patients with SABI.
Section 5	Free space: for additional comments.

### 3.2 Development and pilot experimentation phase

In this phase, intervention tools were designed in accordance with objectives and strategies identified in the preceding phase. An initial experimentation of the products was conducted to study their feasibility and initial acceptability. The working group identified at least three key strengths, providing evidence of the protocol's practicality. This experimentation contributed to the implementation of at least one of the proposed solutions for the challenges identified.

### 3.3 Data analysis

The analysis of the qualitative data was conducted using a thematic analysis methodology (18).

OEQ data were analyzed using a mixed-methods approach; quantitative OEQ data were analyzed via descriptive statistics in Excel, while qualitative responses underwent thematic analysis to extract key insights from the participants.

FG data were transcribed verbatim and analyzed in an Excel spreadsheet using Braun and Clarke's thematic model (18). Thematic analysis was conducted by three members of the research group paying particular attention to relational aspects among the participants and the density of interactions. Socio-demographic information was also verbally requested.

Upon conclusion of the analysis of the FG and OEQ data, the results were compared by all professionals of the working group to identify recurrences and parallels.

With respect to the PO data, the information collected was utilized to gain a better understanding of the context. Its analysis was extemporaneous, with a comparison of the data with that from official documents.

### 3.4 Techniques for improving reliability

To ensure the study's rigor, specific methods from ethnographic studies were adopted, in order to pursue the criteria of credibility, transferability, dependability, and confirmability (19, 20) (Table 1).

### 3.5 Ethical aspects

The present study did not involve any direct clinical intervention on patients by the research group; approval from an ethics committee was not deemed necessary. However, all phases of the project followed the ethical principles of the Declaration of Helsinki (21).

Prior to their involvement, the procedures and methods were explained in detail to potential participants. Verbal informed consent was obtained for the FG and implicitly for OEQ, as participants were informed that continuing with the questionnaire constituted their acceptance of the explained principles of data retention and the purpose of the research.

The complete anonymity of the participating professionals was guaranteed, and direct quotes were included only after further individual authorization (22).

The presence of the researchers in the department was authorized by the host institution.

### 3.6 Funding sources and conflicts of interest

The authors and all members of the research group declare the absence of conflicts of interest. The portion of publication costs not covered by the journal will be sustained by the University of Turin, specifically by the Department of Surgical Sciences, with the support of Prof. Chiara Maria Eandi.

## 4 Findings and discussion

The integration of different methodologies and tools proved effective in answering the research questions, as the actions conducted allowed all the indicators defined in the study to be satisfied (Table 1).

Triangulation of the OEQ and FG thematic analyses revealed highly consistent findings across both populations, which proved extremely useful for an in-depth context analysis to define a joint protocol (Table 3). Both OEQ and FG highlighted the value of orthoptic collaboration, existing systemic barriers, and the need for multidisciplinary rehabilitation. FG participants were available and interested, providing on several occasions the opportunity to discuss and clarify general doubts. Similarly, the inclination toward collaboration and unity within the rehabilitation group is also expressed in the OEQ data.

**Table 3:** Relevant aspects and salient themes recurring in the OEQ and in the FG, analyzed separately and discussed in a plenary session.

Debriefing Meeting
**Professional Involvement.** The professionals involved, both the department staff and the professionals who answered the questionnaire, were available, interested, and confident regarding the topics discussed. In the case of FG, the employees of the neurorehabilitation department were extremely available and interested. On several occasions, the colleagues involved gave us the opportunity to discuss general doubts as well. Regarding the open-ended questionnaire, numerous professionals answered the open-ended questions, motivating their choices, even though it was not mandatory, and expressing positive opinions regarding the joint protocol and working in a team with the orthoptist. **Collaboration:** The professionals involved in both qualitative research interventions aim for collaboration and unity within the rehabilitation group; regarding the open-ended questionnaire, they believe in these values even when they are poorly represented in their workplace **Group Characteristics:** In both analyses, the groups under examination were heterogeneous in terms of age and years of service (experience gained); this aspect is fundamental, especially for the purposes of the study's external and internal validity and its transferability. **Key Roles:** The speech-language pathologist, physiotherapist, and assistant orthoptist in ophthalmology were the three most responsive figures in both analyses (although in a different order); this data indirectly confirms what has been found in the literature: nursing staff in this field tend to remain in the background, as noted by Frantz et al. (2020). The good participation of orthoptists and speech-language pathologists is fundamental since the study, at this stage, aims to build a joint assessment protocol precisely between these two professional figures. **Recurring Themes:** The three main thematic areas of the FG also appear to be preponderant from the analysis of the open-ended questionnaire: the usefulness of collaboration with the orthoptist, the critical issues of the current multi-professional collaboration, and the importance of visual and multidisciplinary rehabilitation indications. The need to share information is highlighted multiple times, for example, through a written record and/or a brief report by each professional who performs assessments and treatments, to be left in the clinical chart and accessible to all staff, without forgetting the training and education of caregivers. **Common Elements:** Common and recurring elements related to the figure of the orthoptist and the visual area of the patient with SABI: A recurring element concerns orthoptic assessment and treatment: although considered fundamental by the majority of professionals, they are often difficult to find. At the same time, the professionals themselves and the demographic analysis conducted on the clinical charts show that information relating to visual skills is often absent or incomplete within the clinical chart. The assessments and treatment of visual disorders do not occur regularly and when they are performed (in case of consultations), they do not follow standardized protocols and/or indications. Often the result of the same is difficult for the rest of the staff to use. Emphasis was placed on finding a “common ground and language” through specific training, particularly for the orthoptist who needs specific training on the patient with SABI, also in order to improve interprofessional communication. • The orthoptist's contribution is particularly significant, especially in cases where a visual channel must be found for an AAC strategy, which cannot be separated from collaboration with the SLPs. • The impact of the visual deficit on the global functioning (including ADLs) of the patient with SABI is recognized. SLPs’ Contribution: Common and recurring elements related to the figure of the SLP and the communicative-linguistic and swallowing area of the patient with SABI: Multidisciplinary collaboration is useful and fruitful, especially with the orthoptist, in the field of AAC. Compared to the figure of the orthoptist, the speech-language pathologist is better integrated into the multidisciplinary rehabilitation teams of patients affected by SABI. However, the rehabilitation figure best integrated into the multidisciplinary work group is the physiotherapist. The impact of communicative-linguistic and swallowing deficits on the global functioning (including ADLs) of the patient with SABI is recognized. The information relating to the speech-language pathology area, and even more so to the physiotherapy area, is very often present in the clinical chart (in a percentage clearly greater than the information relating to the visual sphere). The role of the speech-language pathologist is fundamental for the assessment and treatment of swallowing disorders (controlled management and risk minimization) and communicative-linguistic disorders, and for the identification of the most appropriate communication strategies. The joint orthoptic-speech-language pathology assessment protocol has received positive feedback: The staff of the Neurorehabilitation unit collaborated on its construction, welcoming the proposal with enthusiasm; most of the professionals involved through the open-ended questionnaire believe that this tool would certainly be useful in their own settings as well.

Thematic analysis was performed for each question to outline the professionals' perspectives (Supplementary Material S3). All open-ended questions are included in Supplementary Material S4.

### 4.1 PO and documentary data collection

Analysis of PO and relevant institutional documentation mapped the patient journey within the Neurorehabilitation center. Direct observation revealed a departmental culture that values teamwork and patient-centered care. However, while interprofessional communication was actively sought, it lacked formal standardization. Furthermore, clinical records showed fragmented and inconsistent visual assessment data, which hindered a cohesive multidisciplinary reconstruction of the patient's rehabilitative status.

### 4.2 OEQ

#### 4.2.1 The professionals' experience

Participants unanimously recognized the critical role of visual, cognitive, and communicative impairments in the rehabilitation process. In 34 responses (68%), visual deficits were specifically linked to impaired oculo-manual coordination, reduced independence in Activities of Daily Living (ADL), and increased difficulty in performing speech and physiotherapy exercises. Similarly, cognitive and communicative disorders were found to hinder skill acquisition and socio-relational autonomy, directly impacting the patient's overall quality of life (Themes A1, A4, and A5).

“It influences communicative and motor enabling aspects. For example, knowing if a patient assumes an abnormal head position due to strabismus is fundamental for the choice of postural and communicative aids” (OEQ-12).

“A large part of the information useful for communication passes through the visual channel; often, the visual channel is the only one that can be used to communicate. Furthermore, strabismus, diplopia, and altered perception of spatial relationships globally influence coordination (especially eye-hand coordination, walking) and affect most ADLs” (OEQ-34).

Regarding clinical documentation, information related to visual aspects (Theme A2) is less present in records compared to motor, cognitive, and communicative-linguistic information.

Professional integration within the rehabilitation team varied significantly between roles. The SLP was perceived as effectively integrated by 77.6% of respondents (Theme C4). Key strengths of the SLP role (Theme B2) included the management of communication and swallowing disorders, the provision of personalized treatments for patients and caregivers, and the promotion of cognitive stimulation.

In contrast to other roles, 73.5% of professionals perceived the orthoptist as not effectively integrated into the team (Theme C5). This marginalization was attributed to a general lack of awareness regarding post-acquired visual disabilities and professional isolation.

Noted strengths (27 responses) include the integration of evaluation and treatment of visual disorders, personalized rehabilitation, the provision of strategies and aids for independence in ADLs, consultation to other professionals, and the integration visual rehabilitation with motor and communicative recovery (Themes A3, A4). Specifically, orthoptic insights into saccades or hemianopsia are vital for tailoring communication boards and environmental stimuli (OEQ-102), aiming for the “complete management” of the patient (OEQ-91). Meanwhile, the physiotherapist was unanimously seen as well-integrated (Theme C1), primarily for their role in motor-cognitive functioning and team collaboration (Theme B3).

Overall, the role of the orthoptist presented some positive and negative aspects. The three main strengths were: 1. Relevance of visual deficits on the global functioning of patients is recognized; 2. Collaboration is considered effective for holistic patient management; 3. Teamwork is considered stimulating. Instead, the weaknesses identified for the orthoptist's role are: 1. Limited accessibility to the orthoptic assessment and treatment due to a lack of adequate equipment in the department; B. Orthoptists are considered more of a consultant and not part of the team; C. There is a training gap regarding SABI management.

The clinical benefits that stem from collaborating with an orthoptist are as follows:

- Better adaptation and therefore greater reliability of rehabilitation;
- Support in the choice of appropriate Augmentative and Alternative Communication (AAC);
- Improved intervention on postural compensations;
- Possibility of performing a differential diagnosis (e.g., hemianopsia and neglect, ocular torticollis, etc.);
- Orthoptic rehabilitation and selection of the most suitable aids.

##### 4.2.1.1 Evaluative areas

Among the examples provided (8 responses) on the attention given to different evaluative areas (A. Internal Medicine Stability; B. Basic Vital Functions; C. Communicative-Relational and Cognitive-Behavioral Impairments; D. Sensory-Motor Impairments, Mobility and Transfers; E. Retraining for Autonomy in ADLs; F. Adaptation and Social Reintegration Activities), the only mention of orthoptic and ophthalmological evaluation was as an external consultation.

##### 4.2.1.2 Teamwork

All of the respondents are in favor of working in a multi-professional team and recognize it as an added value for the patient and for the professional (Theme C6). A 63.3% have a constant opportunity to work with at least one other rehabilitation professionals. No negative opinions were reported; the 87.8% recognize that working with another professional figure is “totally useful” for the patient; only 12.2% consider it “partially/quite useful” (Theme C2).

The majority of respondents (85.7%, 42 responses) believe that a shared protocol is “certainly useful” or “could be useful” (4.1%, 2 responses). This data reflects a clear need for standardization and coordination. In 5 answers (10.2%) was reported that shared protocols are already in place, but these do not include close orthoptic-speech therapy collaboration for SABI.

##### 4.2.1.3 Free space

Additional qualitative comments in the “Free Space” section highlighted the potential for the protocol to evolve into an even broader multidisciplinary tool, suggesting the inclusion of occupational therapists to complement the speech therapy and orthoptic integration (OEQ-153).

### 4.3 Focus group

In July 2023, a 60-minute FG was held in a suitable environment within the department. A totale of 11 professionals, including 3 physiotherapists, 3 SLPs, 2 nurses, 2 orthoptists, and a physician specialized in physical and rehabilitative medicine. The group was heterogeneous in terms of age (mean = 40 years, SD = 10 years) and years of service in the field of neurorehabilitation (median of 3 years).

Not all participants contributed with the same frequency. While SLPs (14 interventions) and orthoptists (17 interventions) led the discussion, other roles like physiotherapists and nurses provided support through both verbal and non-verbal reinforcement. Furthermore, the communication style evolved from a hesitant start to an assertive and spontaneous exchange with a transition from specialized vocabulary to colloquial one and a faster pace of turn-taking.

#### 4.3.1 Emergent themes

**Figure 1:**
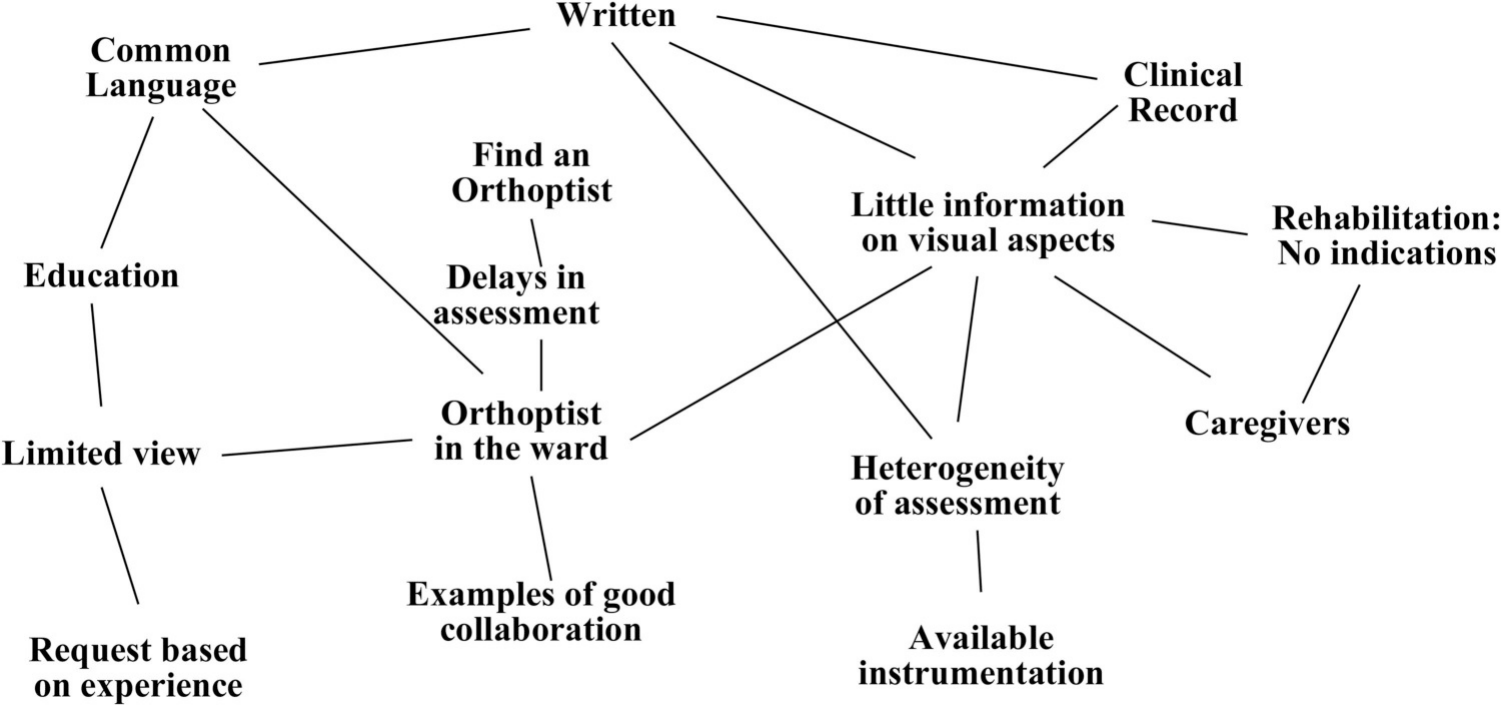
Cognitive map based on thematic analysis of FG.

Through a frequency and logical-connection analysis of the transcripts (Supplementary Material S5), the FG identified three primary thematic areas; This cognitive mapping (Figure 1) provided the empirical foundation for the joint protocol, ensuring that the intervention directly addressed the real-world needs and gaps identified by the clinical team.

##### 4.3.1.1 The usefulness of collaboration with an orthoptist

Key advantages of orthoptic integration (Table 4) included:

- **Increased reliability of SLPs tests and support in choosing AAC**
  “Last year I had a patient who was hospitalized here for a long time who had a big visual problem, and we interfaced with the orthoptist for AAC […]. It was very useful because she gave some indications, and in that case, I was able to see her, and we were there in person. Often, as far as I'm concerned, I happen to see the visit already done […]. [FG-37, L9]”.
  “The orthoptist is very useful to us when performing cognitive and linguistic assessments, they help us because many times you don't know whether the test is affected by language or by visual issues. […]” [FG-60, L10]
  In the focus group, evidence also emerged of implicit theories which lead SLPs to produce biased results.
  *“[*…*] For example, sometimes we project tests onto the wall to make them more accessible to the patient but then you risk invalidating the results themselves*…*” [FG-60, L10]*.
  *“It is always thought that proposing large characters is the best thing to do but it is not: if the patient has a tubular visual field—for example—it is necessary to propose characters and stimuli of reduced size.” [FG-62, O4]*.
- **Better environmental adaptation:**
  “It is important for us to know how to adapt to the environment, how to approach them. If you are certain that a patient cannot do that thing [because they cannot see] and not because they don't want to or are not trying hard enough, it puts me in a completely different position. It would be appropriate to have moments of meeting and discussion to better manage the patient 360°.” [FG-57, F1].
- **Better intervention on postural compensations:**
  “But if there is a combined test it’s better, because very often the awkward head position is a compensation, you know? So if I remove the compensation I will worsen the visual performance.” [FG-48, F1].

**Table 4:** The table shows: advantages and barriers resulting from the collaboration with the orthoptist emerged in the FG, a summary of criticisms of the current collaboration emerged in the FG, a summary of suggested solutions to barriers emerged in FG, and the final report that relates the characteristics of the the joint assessment protocol to the solutions that emerged from the FG.

Collaboration with the orthoptist	
Advantages	Barriers
Greater reliability of speech-language pathology tests	Distance of the service
Greater support in the choice of AAC	Shortage of orthoptic staff
Better adaptation of the environment	Patient transport
Better intervention on postural compensations	Failure to promptly identify cases of visual difficulties: – No professional experience and training; – No “reliability” of the patient with SABI.

Criticisms of the current collaboration	
Who	Solutions
Orthoptist	– Orthoptist in the team – Written summary – Education
Partiality of relevant anamnestic information in the clinical chart	
No diagnostic instruments to objectify bedside assessment	
No standardized assessment procedures	
Common	
Continuity in following the patient → sharing of handovers	
Specific professional knowledge of other disciplines	
Common shared language	
Neurorehabilitation	
Software with information present, but not accessible to everyone	

Solutions	*LG References*	
Comprehensive and usable assessment	59	F8
Shared assessments during handovers so that everyone is correctly informed	63	L9
A tool that leaves a written record	64	F1
A report, without superfluous technicalities, that summarizes skills	65	O4
A clear and accessible summary sheet for every patient	66	I6
Also useful for providing practical information to caregivers for returning home or managing the bedridden relative in a second-level facility	67	F1
In-depth knowledge of basic SABI principles	56	O4
Physical presence of the orthoptist in the ward	66	I6
Network of professionals to involve upon discharge: trained orthoptists to refer patients to	68	M7
On-the-job training in multidisciplinary teams to better understand the complexity of these patients and to facilitate the transfer of information at the interprofessional level; inclusion of the orthoptist within the working group	69	L10
Use of scholarships	71	L10

Solutions	Protocol
A tool that leaves a written record	Structured on an Excel sheet, printable on paper
A clear and accessible summary for each patient	Accessible to all, as it uses software already available in the department (not tied to specific programs) Fillable for each patient
Shared assessments during handovers to ensure everyone is correctly informed	
A report that synthesizes professional expertise without unnecessary jargon	Focuses on function, not just on anatomy-physiology
An understandable and usable assessment	
Useful for providing practical information to caregivers for home discharge or for managing bedridden patients in a second-level facility	Facilitates a quick overview of the patient's overall situation

##### 4.3.1.2 Criticisms of the current collaboration and proposals for possible solutions

Among the felt criticisms, the participants reported several main barriers (Table 4):

- **Service geography:**
  “The patient in question waited two months to come because, put in a wheelchair, he collapsed and, therefore, how was it possible to organize an ambulance transport?” [FG-42, M7]
- **Staff shortages:**
  “It’s a huge problem of people, of spaces… there is no staff to answer the phone. If the orthoptist is there at that moment, they answer; if the shifts are covered on that day, they come for a consultation… the big problem? There are many aspects at stake and there are really few people.” [FG-50, O4]
- **Lack of useful information for orthoptists** in the clinical chart.
  “Since I’ve seen the department’s medical records, what I noticed is the lack of visual information that is collected. This is a given because orthoptists and ophthalmologists are rarely present here, if not for consultation, from what I understand. […]” [FG-22, O4]
- **Lack of standardized tools and common procedures** among consulting orthoptists.
  “[…] And often these assessments are done by different people and not in a standardized way because it also depends on the tools that can be used with the patient you find in front of you. […]” [FG-22, O4].
  “The difficulty is often ours too. It is also difficult for us to find tests because our tests are psychophysical: if we do it today it is different from what I can do tomorrow. However, there are some things, some parameters that can be objectified, but which pass through machines, more complex instruments, or instruments in the development phase, so they cannot be used on the pathological patient.” [FG-28, O4].
- **Difficulty in early identification of patients with potential visual difficulties** for further investigation. This barrier was further specified by the participants, who cited the lack of experience and professional training of non-orthoptist staff in the visual field, and the perceived lack of “reliability” of the patient with SABI in providing consistent responses during standard visual assessments.
  “Sometimes they also happen a little late, because the consultation is requested when the need becomes apparent.” [FG-22, O4].
- **Difficulty in interdisciplinary communication** with complex handovers due to the lack of a common language.
  “Above all, it would be useful for the assessment once performed to be understandable and usable.” [FG-59, F9].
  “[…] Often, as far as I'm concerned, I happen to see the visit already done and often there are acronyms and things that are not explained. Of course, I try to look them up on the internet, but it’s a bit complex. […]” [FG-37, L9].
  “I do see acronyms, “OD/OS etcetera etcetera” and then, I look for it, but having something written down…” [FG-39, L9].
  The concept of a lack of common ground was also reported by the orthoptists.
  “We are not trained for the patient with SABI, we have basic knowledge, but they are limited, even in the field of curricular internships (we almost never see these patients).” [FG-56, O4].

To overcome the identified barriers, the FG participants themselves suggested several key solutions (Table 4):

- Stable inclusion of the orthoptist in the team.
- Using a written form and/or a shared protocol to share an integrated assessment and a common language.
- Sharing written tools and assessments to facilitate handovers.
- Implementing a training program for all staff on the integration of visual aspects.

##### 4.3.1.3 The rehabilitation pathway

The lack of integrated orthoptic support extends even after hospital discharge from the Neurorehabilitation unit. Even when visual deficits are identified, patients and caregivers often find no specialized services in the community [FG-51, M7; FG-52, O3]. This lack of local expertise prevents long-term management of conditions like diplopia, ultimately compromising the patient's quality of life and long-term recovery.

### 4.4 Practical implication

#### 4.4.1 Consistency between OP, OEQ, and FG

The strong activity of SLPs, Physiotherapists, and Orthoptists in the discussions reinforce the relevance of focusing the study on the collaboration between SPLs and orthoptists for the development of the joint protocol. This convergence of opinions between different tools increases the validity of the results and suggests that the problems and proposed solutions are deeply felt in the rehabilitation context. A further significant element that emerged from participation in both the FG and the OEQ is the clear willingness of other professional figures, such as Physiotherapists, Occupational Therapists, and TNPEE, to be involved in the process under examination.

The strong convergence between OEQ and FG findings highlights both systemic barriers and opportunities for optimizing multidisciplinary SABI management. Beyond confirming existing literature (23, 24), our data underscore how visual impairments functionally hinder the recovery of broader rehabilitative skills. This is particularly critical given that clinical guidelines (8, 25) identify the visual channel as a primary support for cognitive-linguistic rehabilitation; any unaddressed visual deficit, therefore, risks compromising the entire therapeutic process.

A primary finding from both OEQ and FG data is the marked underrepresentation of information on visual aspects in clinical charts compared to motor, cognitive, and communicative-linguistic information. This documentation gap reflects the perceived marginalization of the orthoptist within the rehabilitation team. This discrepancy between the recognition of the importance of visual disorders and the poor integration of the dedicated professional leads to the felt need to promote the inclusion of the orthoptist more stably. Professionals have, in fact, articulated the numerous benefits deriving from the integration of the orthoptist, ranging from the assessment and treatment of visual disorders that are often neglected, to support in differential diagnosis, to the optimization of aids and the adaptation of therapeutic strategies for a more holistic and less frustrating approach for the patient. These benefits are also extend to the cognitive domain; our analysis supports recent evidence (13), which suggests that ophthalmological-orthoptic aspects can serve as objective indicators of EFs. This is essential to complement traditional neuropsychological tests that often fail to account for the prevalence of ophthalmological and visual problems in the SABI population, thereby risking a distorted attribution of deficits (11).

Furthermore, as reported by the participants, common post-SABI ocular disorders, including accommodative dysfunction, convergence insufficiency, and visual field deficits (11, 26, 27), influence SLP and Physiotherapy outcomes. Therefore, early identification enables several adaptations, such as optimizing viewing positions based on oculomotor or visual field deficits. It also facilitates the adjustment of stimulus size and contrast to account for altered visual acuity, as well as the implementation of corrective aids (e.g., including glasses, magnifying lenses, or prismatic lenses) for convergence deficits or diplopia (24).

FG analysis identified further concrete barriers to optimal management of visual aspects. Including organizational constraints such as staff shortages and fragmented service distribution. Furthermore, early identification of patients with potential visual difficulties is hindered by the perceived unreliability of SABI patients' symptoms and a lack of specialized visual training among non-orthoptic staff.

Orthoptists expressed a sense of being hindered by the partiality of relevant anamnestic information in the clinical chart, the absence of diagnostic instrumentation to objectify the bedside assessment, and the lack of common standard procedures among all consultants. These confirm a systemic lack of common language; that is a difficulty also felt by the other professionals, especially during handovers. Furthermore, the variability in methods among different orthoptic consultants, or between the consultants and the department, creates inconsistencies in documentation, directly compromising information sharing and continuity of care.

#### 4.4.2 Development and value of the outputs

To optimize multidisciplinary management in Neurorehabilitation, our study analyzed healthcare professionals' experiences to identify key systemic gaps (Table 4).

Findings revealed scarce orthoptic documentation in clinical charts and a lack of professional integration, despite the acknowledged impact of visual disorders on SABI recovery.

These issues are exacerbated by a shortage of diagnostic tools and standardized procedures, even within the orthoptic discipline itself. The direct consequence is a difficulty in creating a common language among professionals, which hinders interdisciplinary communication and complicates handovers.

The combination of evidence from both published literature and the experiences and perspectives of the staff provided the necessary basis for the targeted development of the Joint Assessment Protocol and the Orthoptic Assessment Form.

### 4.5 Joint assessment protocol

The Joint Assessment Protocol directly addresses the need to create a written, clear, shareable, and ICF-based document that can facilitate communication between speech therapists and orthoptists and systematize visual and speech-language assessment. Its flexible architecture fosters a common language and facilitates future integration of other professional roles.

The product consists of two components: Minimal Joint Assessment Protocol (MAP), a paper-based tool for a first overall assessment, and the Global Joint Assessment Protocol (GAP): a comprehensive Excel-based file for longitudinal in-depth analysis (15).

Both have a three-part structure: anamnestic data, consciousness assessment, and joint orthoptic-speech-language assessment stratified by the patient's consciousness and collaboration, based on the Levels of Cognitive Functioning—Revised (LCF-R) (28).

Each section refers to the ICF areas. At least the first two ICF levels are included, with sub-levels for speech-language and orthoptic aspects. The assessment adopts the ICF qualifiers (Likert scale 0–4), with “NA” for “Not Assessable.” This checkbox-based system ensures rapid completion while allowing for longitudinal monitoring of patient evolution. Furthermore, the protocols provide evidence-based suggestions for tests and procedures aligned with departmental practices and literature.

#### 4.5.1 Orthoptic assessment form

The Orthoptic Assessment Form was created to standardize and deepen the orthoptic assessment within the discipline. It includes general patient data, visual symptomatology, and a craniofacial inspection, followed by a comprehensive objective examination of visual and oculomotor functions. The latter section investigates visual acuity, color vision, and stereopsis, alongside reflexive and motor responses such as pupillary light reflex, optokinetic nystagmus, VOR, and visual field extension. It also assesses ocular deviation, extraocular motility, fixation, and the convergence-accommodation-miosis triad, as well as saccades, smooth pursuit, and eye-hand coordination. The form concludes with a concise, multidisciplinary report detailing rehabilitative strategies and clinical advice for the wider healthcare team.

#### 4.5.2 Feedback from the pilot study

The products were created and introduced at the AOU for a preliminary trial. The feedback, consolidated through an audit with SLP, orthoptists, and members of the research group, confirmed their effectiveness in addressing previously identified gaps. The MAP proved highly practical for bedside reporting, while joint assessments enriched the SLP evaluation with new clinical insights. Conversely, the orthoptic assessment benefited from a more ecological perspective during functional daily tasks. This collaborative dynamic also improved the consultants' ability to communicate effectively with patients affected by severe communication disorders.

### 4.6 Limitations and future perspectives

The qualitative nature of the study provided a wealth of detail on the experiences and perceptions of the professionals. But it does not allow for statistical generalizability of the results.

The pilot study represents an initial, uncontrolled application of the tools; thus, the observed benefits require validation through larger, longitudinal studies. Future research should aim to overcome these limitations by integrating additional professional figures, developing specialized visual training for non-orthoptic staff, and evaluating the long-term impact on the efficiency of services and the team satisfaction.

### 4.7 Conclusions

This study aimed to improve the efficiency of multidisciplinary patient care in Neurorehabilitation departments, with particular attention to the integration of visual aspects.

Qualitative investigation via OEQ and FG confirmed that visual impairments significantly impact SABI recovery, yet remain underrepresented in medical records due to poor orthoptic integration and a lack of standardized procedures. These gaps provided the empirical basis for developing the Joint Assessment Protocol (MAP and GAP) and a dedicated Orthoptic Assessment Form. These products were designed to address the expressed needs to systematize visual and speech-language assessment, and to promote more effective communication between SLP and orthoptists, and facilitate a more holistic and patient-centered approach.

The first pilot study indicates that joint protocols enrich diagnostic insights and optimize rehabilitation goal-setting. This work underscores the orthoptist's essential role in the multidisciplinary team.

Scaling these practical solutions through further research and targeted training could significantly enhance clinical efficiency and the quality of care for SABI patients.

## Data Availability

The raw data supporting the conclusions of this article will be made available by the authors, without undue reservation.
