# Supplementary material for: Development and implementation of an integrated orthoptic–speech therapy assessment protocol in neurorehabilitation setting: A community case study
Source: Front Health Serv. 2026 May 20;6:1715851. doi: 10.3389/frhs.2026.1715851 (PMC13230197; doi:10.3389/frhs.2026.1715851)
Supplement: Supplementary file 1 [file Table1.docx]

Supplementary Material

| **Supplementary Material 1.** Grafic description of the analyzed care process. | | | | | | | | |
| --- | --- | --- | --- | --- | --- | --- | --- | --- |
| **SERVICE PRODUCTION SYSTEM** | | | | | |  | **CLIENT** | |
| **CONCEPTS OF THE SERVICE** |  | **CORPORATE MISSION** |  | **INVISIBLE PART** | **VISIBLE PART** |  |  |  |
|  |  |  |  | Auxiliary Structure  Technology and know-how systems  Managers and Executives  Functions and Professionalism | Structure  Operating Systems and Procedures  Organization  Physical Resources and Equipment |  | **CLIENT EXPECTATIONS** | Personal Needs  Previous Experience  Company Image  External Communication  Word of Mouth  Lack of Communication |
|  |  |  |  | **CORPORATE CULTURE FOR THE CLIENT** | |  |  |  |

| **Supplementary Material 2.** Literature search: research questions and search strings. |
| --- |
| **Research Question 1**  What are the effects on resource optimization, quality of rehabilitative care, and improved patient quality of life from a joint multidisciplinary orthoptic and speech-language therapy assessment in adults with Severe Acquired Brain Injury (SABI), compared to standard (monodisciplinary) procedures?  P (Population)   - SABI (severe brain injury, non-progressive severe acquired brain injury) in the post-acute and rehabilitative phase; adults (18+ years); LCF >= 3;   I (Intervention)   - Multidisciplinary orthoptic-speech-language assessment   C (Comparison)   - Assessment according to standard (monodisciplinary) procedures   O (Outcome)   - Resource optimization; improved quality of rehabilitative care; improved patient quality of life.  1. **Research Question 2**   What are the effects on resource optimization, quality of rehabilitative care, and improved patient quality of life from an orthoptic assessment in adults with Severe Acquired Brain Injury (SABI)?  P (Population)   - SABI (severe brain injury, non-progressive severe acquired brain injury) in the post-acute and rehabilitative phase; adults (18+ years); LCF >= 3;   I (Intervention)   - Orthoptic assessment of visual skills   O (Outcome)   - Resource optimization; improved quality of rehabilitative care; improved patient quality of life.  1. **Research Question 3**   What are the effects on resource optimization, quality of rehabilitative care, and improved patient quality of life from a speech-language therapy assessment in adults with Severe Acquired Brain Injury (SABI)?  P (Population)   - SABI (severe brain injury, non-progressive severe acquired brain injury) in the post-acute and rehabilitative phase; adults (18+ years); LCF >= 3;   I (Intervention)   - Speech-language assessment of cognitive and communicative skills   O (Outcome)   - Resource optimization; improved quality of rehabilitative care; improved patient quality of life. |

| **PUBMED** | |
| --- | --- |
| **Speech-language** | ("Brain Injuries"[Mesh] OR acquired-brain-injur* OR severe-brain-injur* OR acute-brain-injur* OR "Cognitive Dysfunction"[Mesh] OR cognitive-dysfunction* OR cognitive-disorder* OR cognitive-decline* OR mental-deteriorat* OR cognition-disorder* OR cognitive-defect* OR cognitive-deficit OR cognitive-disabilit* OR amnestic) AND ("Neurological Rehabilitation"[Mesh] OR neurological-rehabilitat* OR cognitive-training OR brain-training OR cognitive-rehabilitat* OR memory-training OR stroke-rehabilitat* OR neurorehabilitat* OR neuro-rehabilitat* OR neurologic-rehabilitat*) AND ("Speech Therapy"[Mesh] OR "Language Therapy"[Mesh]  OR language-therap* OR speech-therap* OR logopedic-education OR logopedic-training OR speech-education OR speech-training) |
| **Orthoptic** | ("Brain Injuries"[Mesh] OR acquired-brain-injur* OR severe-brain-injur* OR acute-brain-injur* OR "Cognitive Dysfunction"[Mesh] OR cognitive-dysfunction* OR cognitive-disorder* OR cognitive-decline* OR mental-deteriorat* OR cognition-disorder* OR cognitive-defect* OR cognitive-deficit OR cognitive-disabilit* OR amnestic) AND ("Neurological Rehabilitation"[Mesh] OR neurological-rehabilitat* OR cognitive-training OR brain-training OR cognitive-rehabilitat* OR memory-training OR stroke-rehabilitat* OR neurorehabilitat* OR neuro-rehabilitat* OR neurologic-rehabilitat*) AND ("Orthoptics"[Mesh] OR orthopt* OR "Optometry"[Mesh] OR optometr* OR "Vision, Ocular"[Mesh] OR ocular-vision) |
| **Both** | ("Brain Injuries"[Mesh] OR acquired-brain-injur* OR severe-brain-injur* OR acute-brain-injur*) AND ("Speech Therapy"[Mesh] OR speech OR language OR logopedi*) AND ("Orthoptics"[Mesh] OR orthopt* OR optic OR oculomotor OR "Vision, Ocular"[Mesh] OR ocular-vision) |

| **EMBASE** | |
| --- | --- |
| **Speech-language** | ('acquired brain injury'/exp OR acquired-brain-injur* OR severe-brain-injur* OR acute-brain-injur* OR 'cognitive defect'/exp OR cognitive-dysfunction* OR cognitive-disorder* OR cognitive-decline* OR mental-deteriorat* OR cognition-disorder* OR cognitive-defect* OR cognitive-deficit OR cognitive-disabilit* OR amnestic) AND ('neurorehabilitation'/exp OR 'cognitive rehabilitation'/exp OR neurological-rehabilitat* OR cognitive-training OR brain-training OR cognitive-rehabilitat* OR memory-training OR stroke-rehabilitat* OR neurorehabilitat* OR neuro-rehabilitat* OR neurologic-rehabilitat*) AND ('speech therapy'/exp OR 'language therapy'/exp OR language-therap* OR speech-therap* OR logopedic-education OR logopedic-training OR speech-education OR speech-training) |
| **Orthoptic** | ('acquired brain injury'/exp OR acquired-brain-injur* OR severe-brain-injur* OR acute-brain-injur* OR 'cognitive defect'/exp OR cognitive-dysfunction* OR cognitive-disorder* OR cognitive-decline* OR mental-deteriorat* OR cognition-disorder* OR cognitive-defect* OR cognitive-deficit OR cognitive-disabilit* OR amnestic) AND ('neurorehabilitation'/exp OR 'cognitive rehabilitation'/exp OR neurological-rehabilitat* OR cognitive-training OR brain-training OR cognitive-rehabilitat* OR memory-training OR stroke-rehabilitat* OR neurorehabilitat* OR neuro-rehabilitat* OR neurologic-rehabilitat*) AND ('orthoptics'/exp OR orthopt* OR 'optometry'/exp OR optometr* OR 'vision'/exp OR vision OR optic) |
| **Both** | ('acquired brain injury'/exp OR acquired-brain-injur* OR severe-brain-injur* OR acute-brain-injur*) AND ('speech therapy'/exp OR speech OR language OR logopedi*) AND ('orthoptics'/exp OR orthopt* OR 'vision'/exp OR vision OR optic OR oculomotor) |

| **CINAHL** | |
| --- | --- |
| **Speech-language** | ((MH "Brain Injuries+") OR acquired-brain-injur* OR severe-brain-injur* OR acute-brain-injur* OR (MH "Cognition Disorders") OR cognitive-dysfunction* OR cognitive-disorder* OR cognitive-decline* OR mental-deteriorat* OR cognition-disorder* OR cognitive-defect* OR cognitive-deficit OR cognitive-disabilit* OR amnestic) AND ((MH "Rehabilitation, Speech and Language") OR neurological-rehabilitat* OR cognitive-training OR brain-training OR cognitive-rehabilitat* OR memory-training OR stroke-rehabilitat* OR neurorehabilitat* OR neuro-rehabilitat* OR neurologic-rehabilitat*) AND ((MH "Language Therapy") OR (MH "Speech Therapy+") OR language-therap* OR speech-therap* OR logopedic-education OR logopedic-training OR speech-education OR speech-training) |
| **Orthoptic** | ((MH "Brain Injuries+") OR acquired-brain-injur* OR severe-brain-injur* OR acute-brain-injur* OR (MH "Cognition Disorders") OR cognitive-dysfunction* OR cognitive-disorder* OR cognitive-decline* OR mental-deteriorat* OR cognition-disorder* OR cognitive-defect* OR cognitive-deficit OR cognitive-disabilit* OR amnestic) AND ((MH "Rehabilitation, Speech and Language") OR neurological-rehabilitat* OR cognitive-training OR brain-training OR cognitive-rehabilitat* OR memory-training OR stroke-rehabilitat* OR neurorehabilitat* OR neuro-rehabilitat* OR neurologic-rehabilitat* OR (MH "Language Therapy") OR (MH "Speech Therapy+") OR language-therap* OR speech-therap* OR logopedic-education OR logopedic-training OR speech-education OR speech-training) AND ((MH "Optometry") OR orthopt* OR optometr* OR ocular-vision OR optic) |
| **Both** | ((MH "Brain Injuries+") OR acquired-brain-injur* OR severe-brain-injur* OR acute-brain-injur*) AND ((MH "Language Therapy") OR (MH "Speech Therapy+") OR language OR speech OR logopedi*) AND (orthopt* OR ocular OR vision OR optic OR oculomotor) |

| **Supplementary Material 3.** Overall thematic analysis of the OEQ. | | | | | |
| --- | --- | --- | --- | --- | --- |
| **A. VISUAL ASPECTS** | | **B. COGNITIVE-COMMUNICATIVE AND MOTOR ASPECTS** | | **C. TEAMWORK** | |
| **THEME** | **TOTAL** | **THEME** | **TOTAL** | **THEME** | **TOTAL** |
| [A1] Influence of visual deficits on the global functioning of patients with SABI, with a particular impact on: communication, proprioception, movement, and the execution of rehabilitation activities. | 31 | [B1] Information on motor, cognitive, and communicative-linguistic aspects is always present in clinical records. | 47, 41, 38 | [C1] The physiotherapist is clearly well-integrated into the rehabilitation team for patients with SABI, compared to other professional figures. | 48 |
| [A2] Lack of information regarding visual skills in clinical records. | 31 | [B2] Speech therapy evaluation and treatment are fundamental for communicative, cognitive, and swallowing disorders. | 31 | [C2] Holistic and multi-professional management is optimal for patients with SABI. | 43 |
| [A3] Orthoptic evaluation and treatment are often difficult for patients with SABI to access, despite being considered strictly useful. | 27 | [B3] Physiotherapy evaluation and treatment are fundamental for managing motor aspects and autonomy. | 26 | [C3] Need for standardization in the procedures for evaluation and treatment of patients with SABI. | 42 |
| [A4] Need to share information regarding the patient's visual abilities with the working group. | 17 | [B4] Influence of cognitive-communicative and motor deficits on the global functioning of patients with SABI, with a particular impact on comprehension and relationships. | 19 | [C4] The speech therapist is moderately well-integrated into the rehabilitation team for patients with SABI. | 41 |
| [A5] An unrecognized and untreated visual deficit worsens the prognosis (including the recovery of autonomy), and increases disability and frustration. | 8 | [B5] The persistence of cognitive-communicative and motor deficits leads to a worse prognosis and less independence in ADLs. | 10 | [C5] The orthoptist is not effectively integrated into the rehabilitation team for patients with SABI. | 40 |
|  |  | [B6] The persistence of cognitive-communicative and motor deficits leads to a lower quality of life and a worse relationship with caregivers. | 4 | [C6] Communication and collaboration are fundamental elements in the rehabilitation team for patients with SABI. | 26 |

**Supplementary Material 4.** Open-ended OEQ responses with thematic references of the qualitative analysis (Supplementary Material 3).

**How and why does a visual disorder affect?** (34 responses)

1. Dysperceptive deficits, eye-hand incoordination. [A1]
2. It can influence the selection of stimuli and their implementation during treatment. [A1]
3. At the level of visual exploration in terms of visuo-perceptive aspects. [A1]
4. In all phases of recovery, from consciousness disorder to returning to one's social role; furthermore, it is one of the ways to connect with the environment and with others and to interact. [A1] [A5]
5. It affects because the visual channel is fundamental for good interaction with the environment and for carrying out numerous daily activities. [A1] [A5]
6. Vision could be a communicative channel for that patient to show contact with the environment. A visual disorder can create difficulty in performing physical and speech therapy exercises. [A1]
7. It can worsen the patient's cognitive and motor performance. [A1]
8. Increases disability. [A5]
9. In customizing the objectives of the rehabilitation project, specific compensatory strategies that are not always intuitive for the physiotherapist must be used. [A4]
10. It modifies visuospatial perception, interferes with proprioceptive and vestibular abilities, and with the recovery of body image. [A1] [A5]
11. It can increase the perceptive disorder that is already present with the outcome of the brain injury. [A1]
12. It influences communicative and motor enabling aspects. For example, knowing if a patient assumes an abnormal head position due to strabismus is fundamental for the choice of postural and communicative aids. [A1] [A5]
13. Increases disorientation. [A1]
14. It influences the possibility of using communicative and other aids, as well as the recovery of autonomy. [A1] [A5]
15. Increases motor difficulty. [A1]
16. It can influence non-verbal communication and the use of AAC (Augmentative and Alternative Communication) devices. [A1]
17. The alteration of visual ability and perception implies the impairment of a communication channel. [A1]
18. Because it also makes it difficult to work on language rehabilitation. [A1]
19. It influences motor skills, activities of daily living, autonomy, academic and/or work tasks, and communicative skills. [A1]
20. It makes test administration complex and requires adaptation of rehabilitative material. [A1] [A4]
21. It limits potential recovery even in ecological terms (e.g., returning to driving, sports, etc.). [A5]
22. Reading, gaze orientation, AAC. [A1]
23. It influences other skills that the speech therapist evaluates. [A1]
24. Increases disorientation. [A1]
25. Because it influences the perception of the environment and the spatial relationships between the patient's body and the surrounding environment. [A1]
26. It influences perception, balance, the sense of the body in space, etc. [A1]
27. The lack of spatial perception can risk putting the patient in a dangerous condition. It can represent an important source of disorientation. [A1]
28. Removes a channel for receiving external stimuli. [A1]
29. It can impact the prognosis of the patient's level of autonomy and modify their rehabilitation path. [A1] [A5]
30. A perceptual disorder always alters the subsequent processing of stimuli. [A1]
31. Specific deficits that influence cognitive, motor, and communicative recovery. [A1]
32. The vast majority of information passes through the visual channel. [A1]
33. Deficits in comprehension, difficulty expressing oneself and relating. [A1]
34. A large part of the information useful for communication passes through the visual channel; often, the visual channel is the only one that can be used to communicate. Furthermore, strabismus, diplopia, and altered perception of spatial relationships globally influence coordination (especially eye-hand coordination, walking) and affect most ADLs. [A1] [A5]

**How and why does a cognitive and communicative disorder affect?** (29 responses)

1. Inability to independently manage even simple situations. [B4]
2. In terms of learning and transferability of learned skills to different contexts. [B4]
3. It limits the possibilities of recovering ADLs and IADLs, besides making relationships with others and the environment difficult. [B5]
4. The patient's recovery from all points of view cannot disregard good behavioral regulation and minimum cognitive requirements. [B5]
5. Difficulty expressing oneself, difficulty performing exercises, difficulty interacting with the person. [B4]
6. They worsen the patient's recovery prognosis. [B5]
7. Decreases the possibilities of recovery. [B5]
8. It influences the achievement of goals and the rehabilitation prognosis. [B5]
9. It interferes with rehabilitative activity, with the recovery of socio-relational skills, and with compliance. [B5]
10. Comprehension with operators or caregivers could be affected. [B6]
11. It influences the habilitative proposals that can be provided, risking underestimating the patient's actual state of consciousness. [B4] [B6]
12. Inability to communicate which creates frustration. [B4]
13. A cognitive disorder pervasively and with variable degree affects recovery and functional re-education. The communicative disorder limits the patient's possibilities to act on the context and/or understand it, limiting their social participation. [B4] [B5]
14. In learning. [B4]
15. The patient's resources are scarcer. [B4]
16. It alters test administration, adaptation of rehabilitative material, exercise instructions, interface, and compliance. [B4]
17. They pervasively influence both the socio-relational level and the reintegration phase, compromising residual autonomies. [B5]
18. It alters the quality of life. [B6]
19. The goals and tools of the treatment path change. [B4]
20. Increases disorientation and frustration. [B4]
21. Because it influences both the comprehension of instructions, the awareness of one's own disorders and limits, the perception of risk, as well as motor initiative. [B4]
22. It influences comprehension and learning. [B4]
23. A cognitive deficit negatively affects aspects of daily life. The motor sphere and the cognitive sphere are inseparable areas. A communicative deficit has very bad long-term implications also concerning the affective and social sphere. [B4] [B6]
24. It alters the ability to remodel afferences. [B4]
25. It can invalidate rehabilitation, compromising the ability to recover and/or extending the time needed to obtain the maximum possible level of autonomy for the patient. [B5]
26. Often it is the "core" of the problem, not "influencing" it. [B4]
27. Attentional, memory, and executive function deficits can strongly limit the recovery of even mild deficits (anosognosia, difficulty learning compensation strategies, etc.). [B5]
28. Difficulty communicating, difficulty understanding the given answers. [B4]
29. It influences the individual's global functioning, especially if other spheres of the person are also injured. [B4]

**What are the strengths gained from including this figure in the team?**

(Speech Therapist)

1. Inclusion of an expert in communicative and cognitive difficulties capable of evaluating and planning a rehabilitative intervention and of supporting the team and caregivers in communicating with the patient.
2. Better management of the tracheal cannula weaning process and oral feeding. Help in ensuring a suitable environment for the patient for cognitive stimulation (e.g., tools for orientation). Management of communicative aspects.
3. Collaborates in finding a way to communicate with the patient, in trying to orient them in time and space. Works to improve swallowing problems.
4. Greater exchange of information.
5. Holistic management.
6. Recovery of specific functions (swallowing, language) and support in achieving motor and functional goals.
7. Better comprehension of the patient's global functioning, shared goals, and useful information on how to manage communicative aspects/management of ADLs (e.g., feeding). Useful info on prognostic aspects.
8. It helps a lot to deal with cognitive and perceptual disorders and eventual correction, and the swallowing part is then fundamental.
9. Aspects related to feeding and communication.
10. Fundamental in the treatment process.
11. Early and timely management of communicative-linguistic and cognitive aspects to promote the best possible recovery for the patient.
12. Multidisciplinary approach with remarkable results.
13. The speech therapist can identify which communicative strategies the person with SABI can implement, is able to adequately choose AAC aids, and can also deal with swallowing/phonation/breathing disorders.
14. The goals of speech therapy rehabilitation are team goals, for the recovery of the health status of functions that are interdependent.
15. Improving communication.
16. Holistic management and better treatment effectiveness.
17. Possibility of guiding colleagues on how to interact communicatively with the patient, providing communicative aids, having a functional verbal and non-verbal communicative picture of the patient, and structuring the therapeutic setting.
18. An integrated approach between the neuropsychologist and the speech therapist allows for working on the communicative front in a more articulated way, but also for focusing on aspects such as swallowing, pneumophonic coordination, and bucco-facial praxis.
19. Holistic management according to the biomedical model, increased quality of life for the patient.
20. A broader vision of the possibilities and ways to communicate with the patient.
21. Improves the patient's rehabilitation path.
22. Through this figure, all staff are informed about the patient's communicative problems or swallowing difficulties.
23. Their vision and evaluation are essential to understanding the patient's cognitive-communicative level.
24. I believe that the speech therapist is a fundamental figure precisely because there must be a strong interaction between the cognitive and motor fields.
25. Improvement of alternative or compensatory communicative skills.
26. Care of communicative aspects (especially the identification of an adequate communicative channel).
27. Adequate management of communication by all operators, controlled and risk-minimized management of swallowing.
28. Complete management in all its aspects.
29. Punctual evaluation and rehabilitation of cognitive and communicative disorders.

(Orthoptist)

1. Evaluation and treatment of visual disorders not adequately considered.
2. Inclusion of an expert figure in visual rehabilitation who also has the possibility to directly structure and follow the rehabilitative treatment on the patient, as well as providing consultancy to other operators who interact with the patient. [A4]
3. Integration of visual rehabilitation with the motor/cognitive/communicative recovery path.
4. Understanding and resolving/finding solutions when possible for visual deficits.
5. Better treatment of visual disorders.
6. Increased possibilities for recovering global functions.
7. Achievement of specific goals and facilitation of the rehabilitation path.
8. Better understanding of the patient's global functioning, shared goals, and useful information on how to manage communicative aspects/management of ADLs (e.g., walking). Provides useful information to set up the therapeutic path. [A4]
9. The orthoptist does visits outside our context so it's not always possible to get an input.
10. Evaluation of visual aspects that influence the communicative project: for example, based on how they perform saccades or if they have hemianopsia, indications can be given on how the person organizes their gaze, indications can be given on how to organize communication boards, indicate the appropriate stimulus size, where to propose them, and the distance from the interlocutor. [A4]
11. Sharing salient information with the team that definitely influences the rehabilitation of all other aspects. [A4]
12. Facilitates the rehabilitation path.
13. Allows for the identification of visual damage and whether this can negatively affect the use of aids. [A4]
14. Teamwork and eventual visual rehabilitation. [A4]
15. Knowing how to adapt rehabilitative material, understanding which tests can be administered, and understanding how to adapt the therapeutic setting. [A4]
16. Rehabilitating vision, the main source of external information received by our body.
17. I believe they can provide fundamental information for treatment and for the ways of approaching the patient. [A4]
18. Less stress for the patient when they have to undergo investigations and an immediate response, greater adherence to treatment.
19. Certainly their evaluation, essential for me who doesn't know the subject. [A4]
20. In-depth study of the visual deficit.
21. Discrimination between cognitive deficits and pure visual field deficits. [A4]
22. Resolving doubts about the possible presence of spatial neglect or visual disorders, to be able to set up a correct rehabilitative treatment. [A4]
23. Early evaluation of perceptual disorders and effective and early intervention on any perceptual asymmetries (neglect, hemianopsia...). [A4]
24. Complete management in all its aspects.
25. Comprehension of the type of visual disorder the patient may have, tools to overcome the disorder and rehabilitate it. [A4]
26. Punctual evaluation and rehabilitation of visual disorders and higher quality, more personalized multidisciplinary rehabilitation. [A4]

(Physiotherapist)

1. Management of motor aspects and recovery of autonomy.
2. This is the figure who takes care of the patient's autonomy.
3. Care, evaluation, and rehabilitation of motor aspects.
4. Motor recovery, prevention of secondary damage, collaboration with care staff for posture management, training for caregivers.
5. Improving the person's posture by promoting joint mobility and reducing pain. Patient mobilization. Trying to reach the highest level of autonomy for the patient.
6. Intensive treatment of motor disorders.
7. Management of motor problems.
8. Motor and functional recovery.
9. Rehabilitation starts with these healthcare figures: nurse, physiotherapist, speech therapist; without them, there wouldn't even be a start.
10. Stimulation of motor aspects to improve autonomy and motor skills.
11. Fundamental in the path and programming of the rehabilitative intervention. Close collaboration with nurses.
12. Early and timely management of sensory-motor and cognitive aspects to promote the best possible recovery for the patient.
13. Motor coordination with improvements in learning.
14. Motor rehabilitation.
15. Understanding how to mobilize the patient and what materials they can use.
16. Recovery or enhancement of residual motor skills and obtaining the maximum degree of autonomy in daily life activities, generally with positive repercussions on the emotional front.
17. It allows for working on the motor plan; in particular, information on posture is fundamental for the speech therapist's work.
18. Better performance.
19. All staff are informed about the patient's motor difficulties and their level of autonomy in ADLs.
20. The complete speech therapy evaluation.
21. Recovery of residual motor skills and obtaining the maximum degree of autonomy in daily life activities.
22. Motor and cognitive recovery.
23. Increasing the patient's motor skills with a view to greater autonomy.
24. Integration of motor and cognitive aspects in the attainment of goal-directed actions.
25. Complete management in all its aspects.
26. Punctual evaluation and rehabilitation of motor disorders.

**Examples for the entries**

1. The mentioned aspects are discussed, shared, and monitored, and daily treated by the entire team. For social reintegration activities, there are many limits related to the available offers in the territory.
2. Anti-covid swabs, breathing/nutrition, environmental contact, postural alignment, autonomous dressing, phone use.
3. Management of complex situations.
4. A: having an internal medicine doctor would be indispensable, but this figure is no longer on staff.
5. Daily rehabilitative treatments are performed by the speech therapist and PT, with neuro-ophthalmological and orthoptic evaluations twice a month.
6. A. Anti-covid swabs, B. Computerized monitoring of vital functions C. Use of paper communicative aids D. Use of aids for walking recovery E. Occupational therapy activities F. Group activities.
7. A - B: Patients accessing the service have stable vital values C: Mutism from posterior cranial fossa syndrome, vascular lesions, and CNS tumors with the use of AAC, specific speech therapy rehabilitation D: Vascular lesions and CNS tumors, TNPEE and physiotherapy rehabilitation E: paths with educators in a single or small group, paths with occupational therapists F: Adapted sport with Ugi, Acquarella.
8. A. treatment of infections B. Monitoring parameters C. Aids and training for staff and caregivers D. Training for patient and caregivers and evaluation of aid use E. Evaluation of strategies and any aids F. Occupational therapy.

**Free space**

1. Important rehabilitative figures like the occupational therapist are missing from the list; the protocol could be useful!
2. It's the right direction!
3. Thank you for your work.

**Supplementary Material 5.** Transcription of the FG, thematic analysis and additional notes.

| **N°** | **Transcript** | **Theme** | **Observer Notes** |
| --- | --- | --- | --- |
| 1 | Moderator: Good morning everyone, thank you for being here. |  |  |
| 1 | Moderator: Briefly: for those who don't know us, I'm Federica, an orthoptist and Liria's colleague. We are attending the master's degree in Rehabilitative Sciences of Health Professions and we have developed a thesis project that concerns our two professional figures, and more. The objective we have set for ourselves is to improve the rehabilitative care of patients with Severe Acquired Brain Injury (SABI). We thought about this patient population because often visual, cognitive, and linguistic aspects are affected simultaneously. So we set ourselves the goal of building something more standardized, especially for the orthoptist who is often very far from these patients, very little trained, and almost never present in Neurorehabilitation departments. We would like to hear your expert opinion on this topic, on the collaboration of the various professional figures and what, hypothetically, you think of a possible joint assessment and how important it could be in your great daily work. |  | A few participants return the greetings. Very attentive and silent. Someone brought a pen and paper to take notes. |
| 2 | Moderator: We'll say two more technical things and then we'll start. If you all agree, the conversation will be recorded because it's difficult for us to remember everything you say, and since we want to carefully consider all the opinions expressed by each of you, we want to rely on the recording as well. We would like you to discuss the topics we propose. We know about the efforts you are making to be here, so we will try to be brief and not exceed the hour we asked for. We would like you to discuss the topics that will be proposed from time to time. We don't want everyone to agree. Therefore, both positive and negative evaluations and comments are welcome. In the logic of the focus group (FG) - what we are doing today - our task is to facilitate the comparison of opinions, group discussion, and, therefore, we have a few rules. To make everything more fluid, we will call each other by name; obviously, everything is strictly confidential: in the research report we write, no personal references will appear, neither your names nor those of the patients. You must all speak out loud and in turns. For us, this discussion is very useful and formative. |  |  |
| 3 | M7: What do you use to record? |  |  |
| 4 | Moderator: We use two cell phones. We leave one in the center, and the other we place behind. My colleague uses the computer to start taking notes. |  |  |
| 5 | Moderator: The first part is dedicated to presenting the group. You are the chosen group because you have particular expertise on the matter. In turns, let's briefly introduce ourselves, say our name, what our profession is, how many years of service, and... whatever you deem most appropriate to say. |  | A bit of a pause and furtive glances before starting. |
| 6 | O4: Hi. I'm O4. I'm an orthoptist. I recently started dealing with SABI, since I started working in the ophthalmology department at Molinette and through my master's thesis. |  | A bit of a pause before the next person continues. |
| 7 | O3: I'm O3. I'm an orthoptist and I deal with neuro-ophthalmology. Maybe some of you know me from having worked at Molinette a few years ago, and I also deal with orthoptic rehabilitation at UGI. |  |  |
| 8 | I5: I'm I5. I am the nursing coordinator of Neuro. I have been a coordinator for about 4 years; before that, I almost always dealt with dialysis. |  |  |
| 9 | I6: Hi, I'm I6. I'm a neurorehabilitation nurse and I've been in this department for 3 years. |  |  |
| 10 | M7: My name is M7. I am a doctor in the N. department. I have been working here since my last year of specialization school, so it could be... several years [note: laughter], about fifteen. |  |  |
| 11 | F8: Good morning, I'm F8, a physiotherapist at N. I also, I seem to remember, have been here since 2010, so it's been 13 years. |  |  |
| 12 | L10: I'm L10, a speech-language pathologist at N., and I've been working here for a year and a half. |  |  |
| 13 | L9: I'm L9, also a speech-language pathologist. I have been working for six years and for a year and a half with SABI here in Turin. |  |  |
| 14 | L2: I'm L2, I've been a speech-language pathologist at N. since 2013 and at CTO since '96. |  |  |
| 15 | F11: I'm F11, I'm a physiotherapist at the hospital-territory integration, continuity of care. I work here in the Health City at the Health Directorate. |  |  |
| 16 | M7: The NOCC? |  |  |
| 17 | L10: Yes, that's me. I'm doing my thesis on another topic, on the provision of aids, which is also very important for you, but on the 3rd floor, and I'm here mainly for methodological reasons. |  |  |
| 18 | Moderator: Thank you. We propose to start talking about the impact of cognitive-linguistic and visual problems on clinical practice: we ask you to reflect on their impact and correlate them. |  | No one speaks. The moderator asks the question directly. |
| 19 | Moderator: Thinking about your usual clinical practice, what are the most frequent or important critical issues you encounter that are due to these problems? How do they affect the rehabilitation process? |  | No one answers. Encouragement to speak freely. |
| 20 | Moderator: Speak freely, whoever wants to start talking, can. There is nothing right or wrong. |  |  |
| 21 | O4: Having seen the department's medical records, what I noticed is the lack of visual information that is collected. This is a given because orthoptists and ophthalmologists are rarely present here, if not for consultation, from what I understand. And often these assessments are done by different people and not in a standardized way because it also depends on the tools that can be used with the patient you find in front of you. Sometimes they also happen a little late, because the consultation is requested when the need becomes apparent. So that's my point of view as an outsider and just a recent graduate. You who know this type of patient will have another experience, perhaps. | Scarcity of visual information in the medical record, lack of systematicity and standardization, delays in assessment. |  |
| 22 | M7: In my opinion, in recent years, since there is an ophthalmology service at Molinette, it's easier to get an assessment. | Reference to the change that occurred at the university level: years ago the location for the Degree Course in Orthoptics and Ophthalmological Assistance and for the medical specialization in ophthalmology was the Ophthalmological Hospital; it is now all moved to the San Lazzaro Pavilion, so in the Health City. |  |
| 23 | Moderator: Why, how was it before? |  |  |
| 24 | M7: Just to give a historical overview, in recent years, since the ophthalmology service has been at Molinette, it has been easier for us to get an orthoptic assessment of patients because before... they went to the ophthalmology emergency room... so we were talking about an assessment of chronic and stabilized situations. Now the possibility of having an orthoptist in the department and who, in some cases, assesses patients with the speech-language pathologist and the physiotherapist, has already helped us to understand something more. Then, it is true that there are some patients who get better who come from there to do a visual field, but there is always the doubt whether it is hemianopsia or hemi-inattention... and we will see over time. This was certainly a first step. After that, the difficulty - in practice - is in the rehabilitation work. First of all, with some patients where we have the feeling that there is a visual field problem, there are often no communication skills to assess it: the feeling is that then, perhaps, there is a space in the visual field where the patient responds to stimuli and a direction of the visual field where they don't respond, but what about all those patients who are in conditions of minimal responsiveness? | Orthoptist in the department, assessment request based on experience --> not standard. |  |
| 25 | Moderator: The feeling... why? |  |  |
| 26 | M7: Because it, so to speak, remains there and it is difficult to diagnose it because if there is a lack of communication, without perhaps a consolidated yes/no answer... | Difficulty in understanding a real/simulated visual problem from the condition of the poorly responsive patient. |  |
| 27 | O4: The difficulty is often ours too. It is also difficult for us to find tests because our tests are psychophysical: if we do it today it is different from what I can do tomorrow. However, there are some things, some parameters that can be objectified, but which pass through machines, more complex instruments, or instruments in the development phase, so they cannot be used on the pathological patient. | Some orthoptic tests are objectifiable (systematicity) --> standardization. |  |
| 28 | M7: For example, we have tried, several times, to do visual evoked potentials to understand, on patients with a very severe alteration of the state of consciousness, whether the visual one could be an effective stimulation channel. |  |  |
| 29 | O4: Correct. What we assess, however, through the visual potential is the perception that we don't know if it is processed... |  |  |
| 30 | M7: —if it is processed. But if we already know that the information doesn't arrive, that's already information to work on. |  |  |
| 31 | O4: They are done in the clinic [the visual evoked potentials]. It is a very simple instrument. Potentially they could be done anywhere... if we had the instrumentation. O3 could elaborate on the topic of portable electrophysiology. | Importance of VEPs [visual evoked potentials] and portable electrophysiology. |  |
| 32 | O3: Yes... we did a thesis on smart glasses that would allow measuring flash and pattern VEPs (visual evoked potentials) to also assess the discriminatory capacity of the non-compliant patient, developing them thanks to the collaboration with the polytechnic. The problem is that we got stuck with the ethics committee [note: laughter]. We had obtained excellent repeatability of the tracings. It all started from the fact of using them during operations, in neurosurgery. There is something. The problem is that then it gets stuck because it doesn't find a response or interest or funds to continue the evolution. | Note: laughter. |  |
| 33 | O4: The same thing for the visual field. We developed a viewer for the peripheral visual field with the Turin polytechnic, so for gross visual field deficits such as hemianopsia or anyway quadrantopsia, but an instrument cannot be used because it is at the beginning of its development and cannot be brought into the departments for patients. |  |  |
| 34 | Moderator: You can all intervene freely [note: a few seconds pause]. | Note: a few seconds pause. |  |
| 35 | Moderator: Let's move on to another point. As for relationships, collaborations with other professionals on the department's team, but also extra, what is your experience like? Are there analogies or differences? What could be the strengths or critical issues? Is it useful, when...? You can also give very practical examples. |  |  |
| 36 | L9: Last year I had a patient who was hospitalized here for a long time who had a big visual problem, and we interfaced with the orthoptist for AAC because we had done a test with a pointer, but there were so many movements that in the end it wasn't clean and we switched to another instrument. Fortunately, in that case, I had discussed it with the orthoptist because, well, I see things, but it's not my field and I don't know what to do much, you know? It was very useful because she gave some indications, and in that case, I was able to see her, and we were there in person. Often, as far as I'm concerned, I happen to see the visit already done and often there are acronyms and things that are not explained. Of course, I try to look them up on the internet... but it's a bit complex. | Example of virtuous collaboration between orthoptist and speech-language pathologist on the topic of AAC; Not sharing the same professional language. Partiality of the professionals' view. |  |
| 37 | O4: We noticed that. A guide would be needed: the angle of strabismus, ocular parallelism, stereoscopic sense, etc... | Need for training. |  |
| 38 | L9: Yes! I see acronyms, "OD/OS etcetera etcetera" and then, I look for it, but having something written down... | Need for training --> The use of acronyms and abbreviations limits accessibility and hinders understanding. Refers to the acronyms for Right and Left Eye, CT=cover test, etc. |  |
| 39 | Moderator: Thank you for what you said. Any other ideas on the matter? |  |  |
| 40 | L2: For example, with respect to the patients you saw, we realized that already in the first assessment carried out, the visual disorder had a huge impact, and from your visit other problems came out that were not thought to be... yes, it was suspected... from our tests, things had come out that did not agree with neglect and, in fact, there was a campimetric deficit for which the patient was then brought to the site where he had a certain... a visual field exam. He was cooperative, yes, but there was a very great benefit for us, on the precise and combined assessment, in my opinion. | Benefits and unexpected information following the orthoptic assessment and the joint assessment between orthoptist and speech-language pathologist. -> experience does not always clarify. Another example of the benefit of collaboration. |  |
| 41 | M7: The patient in question waited two months to come because, put in a wheelchair, he collapsed and, therefore, how was it possible to organize an ambulance transport? | Non-transportable patients, need to intervene on site (department) [LATENCY]. |  |
| 42 | O4: For us, in this case, from the assessment done here, it was quite obvious what it was about, by making him fixate on targets, or with more gross tests. Then, it was objectified by the methods we use for diagnosis. | From screenings to orthoptic in-depth analyses. Systematicity: it is possible. |  |
| 43 | L2: Then, we trust what the patient gives us, if it is considered reliable, so if he says "my best eye is the right one" and then it's the left one... from there there is also a diplopia... |  |  |
| 44 | O4: The same thing with abnormal head positions— | Physiotherapists and speech-language pathologists discuss PAC (abnormal head positions) of ocular and non-ocular origin. |  |
| 45 | L2: Oh, yes. Here the physiotherapists intervene well. |  |  |
| 46 | O3: Physiotherapists, we want to hear from you! Because we look at the eyes, and we tell patients "go ahead and position yourself like this [note: he bends to one side]... with your head down to your knee, so you don't see double... but then there are so many other problems. Knowing what to correct and what not... the discussion could also be useful for this. Do we force him to stay straight because that is the best position? Then we can give him prisms; or can he maintain that compensation because... maybe he has been compensating for a long time. We can't figure it out alone. | Partiality of the single professional's view; Assessment and elimination of compensation as another example of successful collaboration. | Note: he bends to one side. |
| 47 | F1: But if there is a combined test it's better, because very often the awkward head position is a compensation, you know? So if I remove the compensation I will worsen the visual performance. The problem is intervening late, also on what is a visual disorder, it also means fixing an incorrect attitude that then becomes almost incorrigible because if the patient spends months with his head rotated and tilted because only in this way can he have a visual performance. We would hardly be able to correct that head there, even though the visual performance returns. In that case, perhaps, the collaboration should be anticipated. | Latency. F8 nods, confirms. |  |
| 48 | M7: In this regard, I point out a "very trivial" practical problem: when we make requests for consultation with so much love, it is difficult to have a practical communication channel... then I discovered that there are more propitious time slots for some of you to answer, take the agenda, and schedule the visits. It's not very practical. | Difficulty in contacting the Ophthalmology Department for consultations --> latencies. |  |
| 49 | O4: It's a huge problem of people, of spaces... there is no staff to answer the phone. If the orthoptist is there at that moment, they answer; if the shifts are covered on that day, they come for a consultation... the big problem? There are many aspects at stake and there are really few people. |  |  |
| 50 | M7: After that, another major difficulty is that, after the patient's hospital stay is over, if I have to direct him to do rehabilitation for diplopia or to be followed for this type of problem, there is no response. | Lack of orthoptic rehabilitation services in the area. |  |
| 51 | O3: I deal with children, oncology patients, it's difficult... as for visual rehabilitation there are very few who deal with these activities. For the most part they are private... the observation is true: there is no place where rehabilitation is done for this type of problem, in the public sector... | Lack of orthoptic rehabilitation services in the area. |  |
| 52 | M7: I also report another problem, sorry for interrupting you. Often the patient is happy to pay privately, but since they often don't go home from us, but maybe they go to a 2nd level or, to the Puzzle or other Day Hospitals... if they are not given the indication "you must continue with vision rehabilitation," they don't know what to do. Secondly, it would be interesting to have a series of competent names to do it... | The hospital does not issue rehabilitation instructions. Recurring theme, very felt by the staff. |  |
| 53 | Moderator: Speaking of sharing care, how would you promote collaboration, what strategies would you adopt? What could be the disadvantages or advantages? |  |  |
| 54 | Moderator: Based on what you have brought up today, hypothesizing, therefore, that you need training on visual aspects and the areas under discussion, could it be useful? How would you want it to be structured and what content should it have? |  |  |
| 55 | O4: As an orthoptist, I would say yes. We are not trained for the patient with SABI, we have basic knowledge, but they are limited, even in the field of curricular internships (we almost never see these patients)— | Need for training for orthoptists in the field of SABI. |  |
| 56 | F1: A possible orthoptic/visual anomaly affects everything... on ADLs for example and on care activities, which directly concerns nurses. I imagine that with a significant visual field deficit or if the patient sees double, it is also very difficult to eat from a plate or to wash in front of the sink... It is important for us to know how to adapt the environment, how to approach them. If you are certain that a patient cannot do that thing [because they cannot see] and not because they don't want to or are not trying hard enough, it puts me in a completely different position. It would be appropriate to have moments of meeting and discussion to better manage the patient 360°. | Example of the repercussions of a visual deficit on the patient's global functioning. Another example of collaboration. Undercurrent noise increases. |  |
| 57 | I6: Nods. | Nods. |  |
| 58 | F8: Above all, it would be useful for the assessment once performed to be understandable and usable. | Common language necessary -> training. |  |
| 59 | L10: The orthoptist is very useful to us when performing cognitive and linguistic assessments, they help us because many times you don't know whether the test is affected by language or by visual issues. Let's say the patient has difficulty with visual memory but elements are missing to assess if this is really the case. For example, sometimes we project tests onto the wall to make them more accessible to the patient but then you risk invalidating the results themselves... | Importance of shared orthoptist-speech-language pathologist assessment. Another example: lack of orthoptist training to understand aspects of speech-language pathologist assessment + partiality. Undercurrent noise increases. |  |
| 60 | L9: Exactly, there is a risk that they will be falsified or that we will adapt. | Closer and more intense exchanges. |  |
| 61 | O4: It is always thought that proposing large characters is the best thing to do but it is not: if the patient has a tubular visual field - for example - it is necessary to propose characters and stimuli of reduced size. | Collaboration between professionals to avoid error/bias. |  |
| 62 | L9: But you understand that these tools and assessments should be shared for handovers so that everyone is correctly informed. | Increase systematicity and sharing of joint assessments. |  |
| 63 | F1: We should find a tool to leave a written record because there are many of us, sometimes someone is missing for holidays, illness, patient transfers... | Leave a record and inform everyone of what has been done. F8 agrees, everyone nods. |  |
| 64 | O4: One thing that orthoptists lack is precisely the continuity of care for patients since we deal mainly with outpatients and ophthalmological assistance, so assessments, diagnostic tests, and/or short-term follow-ups. Professionals like O3 who mainly deal with rehabilitation understand this aspect better. At the same time, other rehabilitation professionals may struggle to understand the orthoptist profession because it is very different from theirs. Very often, orthoptic assessments are full of technicalities that only we care about... in essence, a mini report would be needed at the end of the orthoptic assessment that summarizes that patient's visual skills. | Reduced systematicity because the same patient is often followed by different orthoptists --> specific language to communicate with other orthoptists O3 shares. |  |
| 65 | I6: Likewise, the healthcare assistants have their hands full and need the same information transfer as the rehabilitation professionals. One idea could be a clear and accessible summary sheet for each patient and/or a collective meeting moment for the transfer of information and handovers; obviously, this would require the physical presence of the orthoptist in the facility. | Systematicity with a clear summary sheet from the orthoptist. Orthoptist in the department. I5 nods and confirms. |  |
| 66 | F1: In my opinion, it would also be important to pay attention to caregivers: they also need practical information for returning home or managing the bedridden relative in a 2nd level facility... Many times it is very difficult for caregivers to understand the patient's visual difficulty, for example, it has happened to us that we had to explain to a patient's wife how eyeglasses are not always useful for compensating for a deficit of central origin. | Inform and train the family and caregivers on visual aspects. M7 confirms, general expressions of consent. |  |
| 67 | M7: Another point I wanted to make is the difficulty in finding orthoptists and facilities that deal with visual rehabilitation in the area, after discharge. I realize that the hospital does not have the resources, but for the patient, the lack of a professional like the orthoptist is really a serious deficiency... and this must be underlined! Furthermore, if there are things that can be adopted for a better management of the patient, it is good to know them. It would be very useful for us to have references in the area, a network of professionals to involve upon discharge: trained orthoptists to whom to send the patients. | Patient care in the area, after discharge: references are missing! L9 confirms. Recurring theme, very felt by the staff. |  |
| 68 | L10: I was thinking, in this regard, that on-the-job training in multidisciplinary teams could be useful to better understand the complexity of these patients and to facilitate the transfer of information at an interprofessional level... and then including the orthoptist in the work group would really be beneficial in many ways. I think this aspect is emerging a lot today, here. | Need for on-the-job training with the orthoptist + implementation of the team with the orthoptist. |  |
| 69 | M7: We already do it in part, but... let's build something that works better together since you are also full of enthusiasm! It often happens that the patient has to resort to private orthoptists in the area (e.g. Ciriè), I often keep the contact if the patient says they are happy and send other patients residing in that area... this is a small step, but it cannot be the solution! | Construction of a professional network with orthoptists. + willingness to act. |  |
| 70 | L10: Propose on-the-job training with orthoptists through scholarships? | Proposal for training through a scholarship orthoptist. |  |
| 71 | Moderator: Unfortunately, with the current resources, there is not yet the possibility of organizing an orthoptic rehabilitation service within the Health City, but what we want to do with this work is precisely to demonstrate "the need," to make the importance of these aspects just described clear and to propose a possible intervention... in this sense, your opinion will be decisive. Who better than you can give us this information? Having said that, if anyone wants to add anything, feel free to do so... | Orthoptic rehabilitation clinic: future perspective?: example of future collaboration. |  |
| 72 | All: Nothing... all clear... thank you. |  |  |
| 73 | Moderator: Before we leave, I would like to summarize the situation a bit; briefly, what we talked about. We touched on several points, correct me if I'm wrong. The main ones that were called into question were the lack of common ground among orthoptists, who to date do not have a standardization of assessment, but also among the various healthcare figures, in the sense that the language of orthoptists is very specific and appears cryptic to the many who are not familiar with it. However, this happens when you have the opportunity to request a consultation with ophthalmology. It's true that over the years the situation has improved, but more could be done, right? I say this because you said that to get in touch with the department you use the knowledge acquired through experience and you manage, more or less, to contact the colleagues, the few colleagues there are. The lack of orthoptists is another topic you brought up today. There is a particular lack of colleagues who deal with rehabilitation, even if, currently, in the scientific landscape, more and more tools are starting to appear that could help both in assessment and in rehabilitation. The situation is more dramatic when patients leave the department because it is also difficult to give them directions. The colleagues who saw our protocol hypothesis live found it a clever thing, right? Because it shortens the time for a consultation, especially for non-transportable patients, because orthoptist colleagues can use the skills of speech-language pathologist colleagues to relate to patients, who are not so clean from a communication point of view, but also the speech-language pathologists - and we hope the physiotherapists and nurses and doctors, in the future - benefit from the co-presence as they can not only bypass the search for acronyms, but also fully and better understand the meaning of these. Furthermore, the orthoptist's indications seem useful to reduce the implicit theories of other healthcare professionals: "the bigger it is, the better it is" and to understand for which patients, in reality, it is necessary to increase the dimensions of the tests or of the paper material in general. The written record is useful especially for continuity, in the sense that where there is not just one healthcare professional, in a single-profession setting, and there is a need to leave handovers... all the advantages of the joint assessment are lost because not the entire department is present. Did we capture the right things? |  |  |
| 74 | F1: I have a question. Where is the physiotherapist's part? | Collaboration with physiotherapists + willingness to act. F1 viewed the protocol left available by the observer shortly before. |  |
| 75 | Moderator: It's not there. In the sense that this is a limitation of this current phase. Being a pilot and having, for now, only speech-language pathologists and orthoptists available, all the other parts have not been included because obviously we don't have the skills, but don't worry! We have also thought about how to implement it in the future, if you think it can be a useful tool. |  |  |
| 76 | F1: So, how do we get involved? |  |  |
| 77 | Moderator: I really appreciate this proactivity! Nothing strictly planned, but one idea could be to carry out the project through the three-year degree theses. We are now dealing with the more organizational part with the master's degree. So the more purely clinical aspects would remain uncovered, and therefore the three-year degree could help us. Could that be an idea? |  |  |
| 78 | F1: Nods. | F1 nods. |  |
| 79 | Moderator: The speech-language pathologist colleagues have our contact information. For any doubts, ideas... anything, you can call or send us an email. Does anyone else want to add anything? [Silence] |  |  |
| 80 | Moderator: So, we can conclude our meeting. Thank you all for the important contribution and, hoping not to have taken up too much of your time, see you soon! |  |  |
| 81 |  |  | At the end they stay to chat and clarify aspects of the entire project. |
